# Resistance of *Leishmania (Viannia) braziliensis *to nitric oxide: correlation with antimony therapy and TNF-α production

**DOI:** 10.1186/1471-2334-10-209

**Published:** 2010-07-15

**Authors:** Anselmo S Souza, Angela Giudice, Júlia MB Pereira, Luís H Guimarães, Amelia R de Jesus, Tatiana R de Moura, Mary E Wilson, Edgar M Carvalho, Roque P Almeida

**Affiliations:** 1 Serviço de Imunologia, Hospital Universitário Prof. Edgard Santos, Universidade Federal da Bahia, Salvador, Bahia, Brazil; 2 Universidade Federal de Sergipe, Aracaju, Sergipe, Brazil; 3 Departments of Internal Medicine, Microbiology and Epidemiology, University of Iowa and the VA Medical Center, Iowa City, IA, USA

## Abstract

**Background:**

Nitric oxide (NO) produced in macrophages plays a pivotal role as a leishmanicidal agent. A previous study has demonstrated that 20% of the *L. (V.) braziliensis *isolated from initial cutaneous lesions of patients from the endemic area of Corte de Pedra, Bahia, Brazil, were NO resistant. Additionally, 5 to 11% of the patients did not respond to three or more antimony treatments" (refractory patients). The aim of this study is to investigate if there is an association between the resistance of *L. (V.) braziliensis *to NO and nonresponsiveness to antimony therapy and cytokine production.

**Methods:**

We evaluated the *in vitro *toxicity of NO against the promastigotes stages of *L. (V.) braziliensis *isolated from responsive and refractory patients, and the infectivity of the amastigote forms of these isolates against human macrophages. The supernatants from *Leishmania *infected macrophage were used to measure TNF-α and IL-10 levels.

**Results:**

Using NaNO_2 _(pH 5.0) as the NO source, *L. (V.) braziliensis *isolated from refractory patients were more NO resistant (IC50 = 5.8 ± 4.8) than *L. (V.) braziliensis *isolated from responsive patients (IC50 = 2.0 ± 1.4). Four isolates were selected to infect human macrophages: NO-susceptible and NO-resistant *L. (V.) braziliensis *isolated from responsive and refractory patients. NO-resistant *L. (V.) braziliensis *isolated from refractory patients infected more macrophages stimulated with LPS and IFN-γ at 120 hours than NO-susceptible *L. (V.) braziliensis *isolated from refractory patients. Also, lower levels of TNF-α were detected in supernatants of macrophages infected with NO-resistant *L. (V.) braziliensis *as compared to macrophages infected with NO-susceptible *L. (V.) braziliensis *(p < 0.05 at 2, 24 and 120 hours), while no differences were detected in IL-10 levels.

**Conclusion:**

These data suggest that NO resistance could be related to the nonresponsiveness to antimony therapy seen in American Tegumentary Leishmaniasis.

## Background

Leishmaniasis is a parasitic disease considered a major public health problem affecting 88 countries throughout Europe, Asia, Africa and America, with an annual incidence of 1 to 1.5 million of cases, and 350 million exposures according to the World Health Organization. Specifically in Brazil, the Ministry of Health reports an annual incidence of around 28.000 cases. American Tegumentary Leishmaniasis (ATL) presents with a spectrum of clinical manifestations, including cutaneous (CL), mucosal (ML), disseminated (DL) and diffuse cutaneous leishmaniasis (DCL). The major species that cause ATL in the New World are *L. (Viannia) braziliensis*, *L. (V.) guyanensis*, *L. (Leishmania) amazonensis *and *L. (L.) mexicana *[1]. In the Northeast region of Brazil, CL, ML and DL are caused most often by *L. (V.) braziliensis*; and DCL is most often caused by *L. amazonensis *in the New World [2]. The typical clinical manifestation of cutaneous leishmaniasis (CL) is a single ulcerated lesion with elevated borders, frequently located on the inferior limbs [3]. ML is a destructive disease that predominantly affects the nasopharynx. It is most common in the areas of *L. (V.) braziliensis *transmission and usually occurs months or years after CL [4]. DL is characterized by multiple pleomorphic acneiform, papular or nodular cutaneous lesions in two or more noncontiguous areas of the body [5].

*Leishmania *is a digenetic protozoa with two different life forms: promastigotes and amastigotes. Flagellated promastigotes replicate and mature to the infectious metacyclic form in the gut of sand flies. Promastigotes are transmitted to mammalian host by the bite of infected sand fly vector, which injects parasites into the host skin where they undergo facilitated phagocytosis by a macrophage and subsequently transform to obligate intracellular amastigotes [6,7]. Murine models have taught us that type 1 cell-mediated immune responses are key to immune protection against *Leishmania *spp. infections [8]. Interleukin (IL)-12 is an important cytokine that is produced by macrophages early in the infection. IL-12 induces interferon-gamma (IFN-γ) production by the T *helper *1 (T_H_1) and NK cells [9]. In murine systems, IFN-γ has been shown to act in synergy with another macrophage derived cytokine, tumor necrosis factor alpha (TNF-α), in activating macrophages and leading to the expression of inducible nitric oxide synthase (iNOS). iNOS catalyzes the synthesis of nitric oxide (NO) from arginine, a potent microbicidal agent that leads to killing of intracellular parasites and other microbes [10-12]. Our laboratory has described some *L. (V.) braziliensis *strains with resistance to NO Moreover, there is an association between the size of the initial cutaneous lesion of ATL and NO susceptibility of the parasite isolate *in vitro*. Furthermore, patients infected with NO-resistant *L. braziliensis *presented with larger cutaneous lesions than patients infected with NO-susceptible *L. braziliensis *[13].

The pentavalent antimonials (Sb^V^) meglumine antimoniate and stibogluconate have been the drugs of choice to treat leishmaniasis for decades [14]. The mechanism of Sb^V ^action is unclear. A general consensus is that Sb^V ^acts upon several targets in the parasite, including inhibition of parasite glycolysis, fatty acid beta-oxidation and ADP phosphorylation [15-17]. Sb^V ^is most like reduced by the host cells to the trivalent form prior to its intracellular action (Sb^III^) [18]. *L. infantum *axenic amastigotes have been shown to undergo Sb^III^-mediated DNA fragmentation, suggesting that apoptosis may participate in the mechanism of antimonial action [19]. Moreover, treatment of visceral leishmaniasis with pentavalent antimonials induces reactive oxygen species and NO generation in *L. donovani*-infected macrophages [20]. The emergence of antimony-resistant (SbR) visceral leishmaniasis (VL) in various parts of the world [21] has severely compromised the ability to control the disease. At the Corte de Pedra Health Post, located in an endemic area for cutaneous leishmaniasis situated in the southeast region of the state of Bahia, Brazil, the incidence of the disease is 8,1/1000 habitants. A study defining phenotypic variations of parasite isolates at the point of clinical diagnosis could allow us to predict the evolution and the outcome of disease. Therefore, we evaluated phenotypic differences amongst *L. (V.) braziliensis *isolates from patients who did or did not respond to antimony therapy. Our data suggests that NO resistance could be related to the nonresponsiveness of American Tegumentary Leishmaniasis to antimony therapy.

## Methods

### Parasites and Patients recruiting

The patients described in this manuscript are from the endemic area of Corte de Pedra. Every 15 days our group travels to this population to treat leishmaniasis and other diseases. Patients are submitted for routine diagnostic evaluations that include parasite isolation and blood collection for serological purposes. All human subjects were briefed on procedures and signed informed consent documentation. All work with human subjects was carried out under Maternidade Climério de Oliveira Ethical Committee approval number 5/2006. *L. (V.) braziliensis *parasites were obtained by needle aspiration of the skin lesions in patients with ATL before therapy. Parasites were speciated by isoenzyme electrophoresis and monoclonal antibodies at the Departamento de Bioquímica e Biologia Molecular, Instituto Oswaldo Cruz, Fiocruz, Rio de Janeiro, Brazil [22].

### Responsive and refractory patients

Subjects with ATL were identified and diagnosed at the Corte de Pedra Health Post, Bahia, Brazil. We analyzed 1640 cutaneous leishmnaisis cases from Corte de Pedra, between 2001 and 2003, and found that 5% of the patients needed 3 courses of antimony treatment to heal their lesions, and 11% of the CL cases were refractory to more than 3 courses of antimony treatment. For this present study, patients who healed their lesions with one course of antimony therapy (20 mg Sb^V^/kg/day during twenty days) were considered responsive patients. Patients who received three or more courses of antimony therapy were considered refractory patients. The interval between the courses of treatment was 60 days. Sixteen parasite isolates were studied in total: eight were isolated from responsive patients (Table 1) and eight were isolated from refractory patients (Table 2).

**Table 1:** Optical density of *L. (V.) braziliensis *isolated from responsive patients to antimony therapy exposed to NaNO_2_

Isolates	**NaNO**_**2 **_**(mM)**							
	0	0.25	0.5	1	2	4	8	16
14048	0.401	0.194	0.214	0.206	0.095	0.086	0.071	0.089
14207	0.397	0.361	0.330	0.350	0.242	0.106	0.094	0.082
14806	0.301	0.379	0.339	0.186	0.086	0.044	0.019	0.005
**15171**	**0.401**	**0.316**	**0.238**	**0.165**	**0.088**	**0.056**	**0.040**	**0.045**
**15404**	**0.585**	**0.459**	**0.353**	**0.385**	**0.383**	**0.208**	**0.174**	**0.178**
15468	0.493	0.312	0.232	0.241	0.259	0.109	0.085	0.063
15492	0.396	0.321	0.237	0.228	0.098	0.071	0.047	0.059
15826	0.296	0.125	0.149	0.128	0.120	0.084	0.081	0.082

Values represent means from three experiments for each parasite isolate. Bold face is used to mark the isolates chosen for further studies, illustrated in Figure 2.

**Table 2:** Optical density of *L. (V.) braziliensis *isolated from refractory patients to antimony therapy exposed to NaNO_2_

Isolates	**NaNO**_**2 **_**(mM)**							
	0	0.25	0.5	1	2	4	8	16
**393**	**0.531**	**0.534**	**0.585**	**0.614**	**0.536**	**0.458**	**0.366**	**0.283**
14119	0.474	0.354	0.387	0.358	0.230	0.163	0.114	0.121
14758	0.436	0.367	0.355	0.389	0.353	0.228	0.145	0.138
14808	0.451	0.447	0.517	0.408	0.341	0.227	0.171	0.138
14933	0.475	0.428	0.437	0.353	0.192	0.168	0.135	0.154
15028	0.420	0.294	0.383	0.415	0.244	0.122	0.120	0.104
15226	0.409	0.412	0.370	0.432	0.327	0.223	0.133	0.138
**15344**	**0.382**	**0.232**	**0.171**	**0.249**	**0.183**	**0.078**	**0.053**	**0.056**

Values represent means from three experiments for each isolate. Bold face is used to mark the isolates chosen for further studies, illustrated in Figure 3.

### Isolation and cultivation of *L. (V.) braziliensis*

Parasites isolates *L. (V.) braziliensis *were initially cultivated in tubes with biphasic medium (NNN) consisting of rabbit blood agar overlaid with liver infusion tryptose (LIT), supplemented with 10% heat inactivated fetal bovine serum medium (Sigma Chemical Co., St. Louis, MO). Following isolation, these parasites were cryopreserved. After selection for the present study, parasite cultures were expanded in Schneider's insect medium (Sigma) pH 7.2 supplemented with 10% fetal bovine serum (FBS) and 2% human male urine at 25°C (complete Schneider medium).

### Evaluation of NO susceptibility of *L. (V.) braziliensis *promastigotes by MTT assay

The virulence of *Leishmania *spp. is highest in the stationary phase, a growth phase that is enriched for the infectious metacyclic stage of the organism. The sensitivity of MTT uptake assays, however, is best using log phase parasites. We previously reported that MTT assays of oxidant sensitivity of log phase cultures correlates with oxidant sensitivity and virulence of the same isolate when it reaches stationary phase growth [23,24].

Two hundred μL of *L. (V.) braziliensis *promastigotes isolated from responsive (n = 8) or refractory patients (n = 8) in log phase growth at 2.5 × 10^7 ^parasites/mL in Hanks' balanced solution (HBSS, Sigma, pH 5.0) were exposed to dilutions of NaNO_2 _(NO donor) from 0 to 16 mM. After 4 hours at 25°C, plates were centrifuged and parasites were incubated with 5 mg of MTT/mL [3-(4,5-dimethylthyazol)-2,5-diphenyltetiazoliumbromide] at 25°C for 4 hours. Reactions were stopped with 0.04 N HCl in isopropanol and conversion of yellow MTT to purple formazan, indicating mitochondrial metabolism, was detectable at 540 nm. The percentage of viability was calculated by comparison to MTT concentrations in wells without NaNO_2 _[23]. At least 3 separate NO toxicity assays were performed on each parasite isolate.

### Macrophages culture

Peripheral blood mononuclear cells (PBMCs) were isolated from six different healthy human donors. Briefly, heparinized blood was diluted 1:2 with 0.15 M NaCl, and separated on a Ficoll Hypaque gradient (LSM; Organon Teknika corporation, Durham, NC, USA). Mononuclear cells were collected from the interface between the plasma and the Ficoll. Washed mononuclear cells were suspended in RPMI 1640, 10% heat inactivated AB serum (Sigma), penicillin/streptomycin (complete medium) (Gibco BRL, Grant Island, NY). One × 10^6 ^cells in 200 μL allowed to adhere to 8-well Lab Tek plates for 2 hours at 37°C, 5% CO_2_. Non-adherent cells were removed by rinsing, and the resultant adherent monocytes were allowed to differentiate to monocyte-derived macrophages over six days at 37°C in 5% CO_2_.

### Macrophage infections

Macrophages were infected by using four of the above described *L. (V.) braziliensis *promastigotes in the stationary growth phase, 2 isolated from antimony responsive patients (LTCP 15171 and 15404) and 2 isolated from antimony refractory patients (LTCP 393 and 15344). Growth curves of these *L. braziliensis *isolates were performed and no statistically significant differences were observed between the isolates used for macrophage infection. Promastigotes were grown to the stationary phase, washed by centrifugation, suspended in RPMI 1640 and used to infect macrophages from healthy donors. In each experiment, the macrophages were infected with NO susceptible and NO resistant parasites. Care was taken to infect macrophages from the same individual with NO-resistant and NO-susceptible strains. The infections were done at a parasite/macrophage ratio of 5:1 for 2 hours at 35°C in 5% CO_2_. Extracellular parasites were then removed by gentle washing, and infected macrophages were maintained for an additional 2, 24, 72 and 120 hours. Conditions did or did not contain LPS (100 ng/mL) plus IFN-γ (10 ng/mL), added at thirty minutes before the infection. Cells were stained with Giemsa and the infection levels were enumerated microscopically. Each isolate was tested in at least three separate assays. Macrophages from the six healthy donors were used for the infection assays by all the selected isolates. Although the number of parasite samples selected for macrophage infections is small, these data clearly characterize the differences in the behavior between the parasites with different to NO susceptibilities previously described by our group [13]. The proportion of infected cells and the number of parasite/100 macrophages were enumerated by three independent observers, blinded to the experimental conditions.

### Cytokine determination

The supernatants from cultures were harvested at 2, 24, 72 and 120 hours post infection and stored at -20°C for TNF-α and IL-10 determination by sandwich ELISA technique (TNF-α: R&D Systems, Minneapolis, MN; IL-10: BD-Pharmingen). A standard curve was generated using recombinant TNF-α and recombinant IL-10 to express the results in pg/mL.

### Statistical analysis

Mann-Whitney test was used to compare susceptibility/resistance to NO between *L. (V.) braziliensis *isolates.

One-way ANOVA with Tukey's test to compare the levels of cytokines released from infected macrophages.

Kruskal-Wallis test, Dunn's multiple comparison Test was used to compare the means obtained from infection and multiplication of *L. (V.) braziliensis *in human macrophages.

The analyses were done on GraphPad Prism 3.03 software (San Diego, CA, USA).

A p value < 0.05 was considered as the cutoff for statistical significance.

## Results

### *L. (V.) braziliensis *isolated from refractory patients were more resistant to NO than *L. (V.) braziliensis *isolated from responsive patients

**Figure 1:**
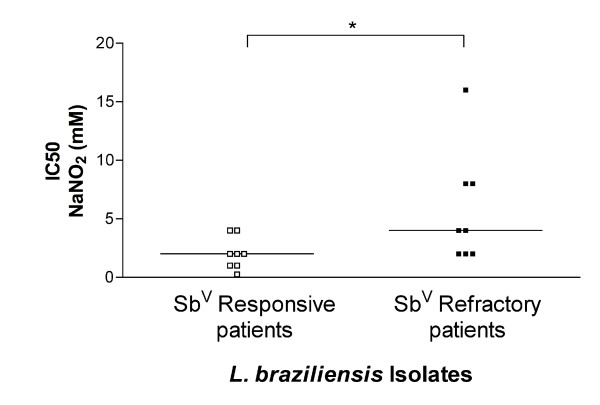
**IC50 of NaNO_2_**. *L. (V.) braziliensis *isolated from antimony-refractory patients were more resistant to the NO-generating compound NaNO_2 _than *L. (V.) braziliensis *isolated from responsive patients. Eight isolates each from refractory or responsive subject were tested for susceptibility or resistance to NaNO_2 _using the MTT assay. Lines mean median (* = p = 0.038, Mann-Whitney test). Lb = *L. (V.) braziliensis*.

We compared the resistance to NO against the responsiveness to antimony therapy of *L. (V.) braziliensis *isolates from patients. We investigated the NO toxicity by measuring the viability of log phase organisms exposed to a gradient of the NO-generating agent NaNO_2_. Viability was measured according to the ability of the parasites to convert MTT to formazan, a measure of mitochondrial activity (Tables 1 and 2). *L. (V.) braziliensis *isolated from antimony-refractory patients were more resistant to NO *in vitro *than *L. (V.) braziliensis *isolated from antimony-responsive patients. The average IC50 (50% inhibitory concentration) was 2.0 ± 1.4 mM NaNO_2 _for isolates from susceptible, compared to 5.8 ± 4.8 mM NaNO_2 _for isolates from resistant subjects (Figure 1). Defining 4 mM NaNO_2 _as intermediate and > 4 mM NaNO_2 _as resistant, only 2 of the 8 isolates from antimony-responsive subjects were intermediate and 6 of the 8 were susceptible. This is in contrast to isolates from antimony-resistant subjects, amongst which 5 of the 8 isolates were either intermediate or resistant and only 3 of 8 isolates were susceptible. These data suggest that NO-resistance might be associated with some cases of resistance to antimony therapy.

### Infection of human macrophages with *L. (V.) braziliensis *isolates from patients that were responsive or refractory to antimony therapy

**Figure 2:**
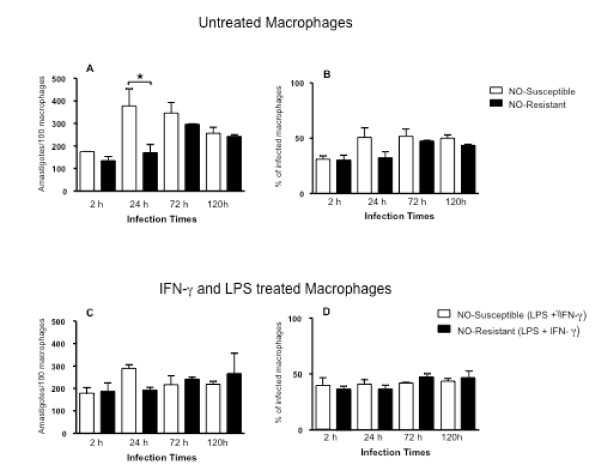
**Infection of human macrophages with *L. (V.) braziliensis *(NO-susceptible - open bars and NO-resistant - closed bars) isolated from patients who responded to antimony therapy**. *A *and *B*, Monocyte-derived macrophages were infected with *L. (V.) braziliensis *promastigotes (5:1 ratio). At 2, 24, 72 and 120 h of culture(*A) *the number of intracellular amastigotes and (*B) *the percentage of infected cells was quantified microscopically as described in *Methods*. (*C) *and (*D) *Monocyte-derived macrophages were treated with LPS (100 ng/mL) plus IFN-γ (10 ng/mL) thirty minutes before infection with *L. (V.) braziliensis *promastigotes. At 2, 24, 72 and 120 h of culture, (*C) *the number of intracellular amastigotes and (*D*) the percentage of infected cells was quantified microscopically. Each bar represents the mean ± SEM infection level in MDMs from three donors (Kruskal-Wallis test, Dunn's multiple comparison Test, * = *p *< 0.05).

To assess whether macrophage infection was influenced by NO resistance and/or antimony responsiveness, we selected four of the above isolates, one susceptible and one intermediate-to-resistant each from the antimony-responsive and the antimony-refractory groups [LTCP 15344 (NO-susceptible) and LTCP 393 (NO-resistant) isolated from refractory patients; LTCP 15171 (NO-susceptible) and LTCP 15404 (NO-resistant) isolated from responsive patients]. Parasites were used to infect human monocyte-derived macrophages (MDMs) from three different healthy donors. After 2, 24, 72 or 120 hours of infection the parasite load was quantified microscopically to assess entry and survival in macrophages. Data using isolates from antimony-responsive patients are shown in Figure 2. After 24 hours of infection, NO-susceptible *L. (V.) braziliensis *infected more macrophages than the NO-resistant parasites (Figure 2A). However, at 120 hours the two isolates had similar levels of infection. When expressed as the percentage of infected macrophages, there was a trend toward increased infection by NO-susceptible *L. (V.) braziliensis *which did not reach statistical significance (Figure 2B). To evaluate whether NO resistance is manifested by resistance to macrophage leishmanicidal activity, we infected macrophages with *L. (V.) braziliensis *isolated from responsive patients after LPS and IFN-γ (Figure 2C and 2D). There was little difference between the infection levels at any time point, suggesting that NO-resistance or susceptibility does not make these *L. (V.) braziliensis *isolates responsive to leishmanicidal activity by MDMs exposed to strong activators of classical activation.

**Figure 3:**
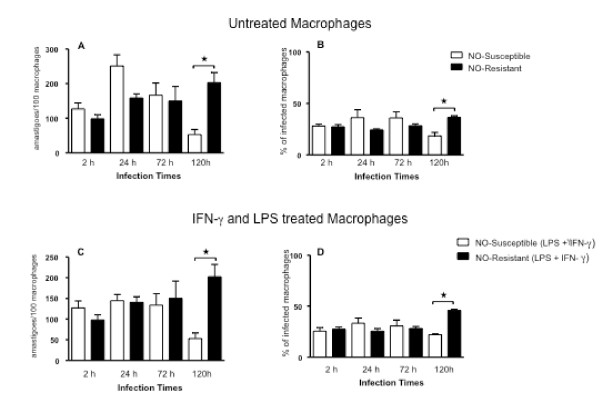
**Infection of human macrophages with *L. (V.) braziliensis *(NO-susceptible - open bars and NO-resistant-closed bars) isolated from patients who were refractory to antimony therapy**. (*A) *and (*B) *Monocyte-derived macrophages were infected with *L. (V.) braziliensis *promastigotes (5:1 ratio). At 2, 24, 72 and 120 h of culture the number of intracellular amastigotes(A) and the percentage of infected cells (B) was quantified microscopically as described in *Methods*. (*C) *and (*D) *Monocyte-derived macrophages were treated with LPS (100 ng/mL) plus IFN-γ (10 ng/mL) thirty minutes before the infection with *L. (V.) braziliensis *promastigotes. At 2, 24, 72 and 120 h of culture, (*C) *the number of intracellular amastigotes and (*D*) the percentage of infected cells was quantified microscopically. Each bar represents the mean ± SEM parasite loads in MDMs from three donors (Kruskal-Wallis test, Dunn's multiple comparison Test, * = *p *< 0.05).

MDM infection with *L. (V.) braziliensis *isolated from antimony-refractory patients showed similar results. The NO-susceptible parasites infected more macrophages at 24 hours of infection compared to the NO-resistant parasites, although the difference was not statistically significant (Figure 3A-B). In contrast to isolates from antimony-responsive subjects, after 120 hours of infection NO-resistant *L. (V.) braziliensis *from antimony-refractory patients achieved a significantly higher burden of infection than NO-susceptible isolates (53 ± 14 amastigotes/100 macrophages). The addition of LPS plus IFN-γ did not affect the results (Figures 3C-D). Overall these results indicate that NO-susceptible isolates achieve a high level of initial infection but are rapidly killed over 120 hrs, whereas NO-resistant isolates either maintain the original infection level (Figure 2) or increase infection (Figure 3).

### TNF-α production is not induced in macrophages infected with NO-resistant *L. (V.) braziliensis*

TNF-α is a proinflammatory cytokine important for the intracellular control of *Leishmania *infection [11]. We evaluated whether TNF-α production is impaired during infection with NO-resistant (LTCP 393 and LTCP 15404) compared to NO-susceptible (LTCP 15171 and LTCP 15344) *L. (V.) braziliensis *isolates from antimony-refractory or antimony-responsive patients. TNF-α levels were measured in supernatants from infected macrophages cultures.

**Figure 4:**
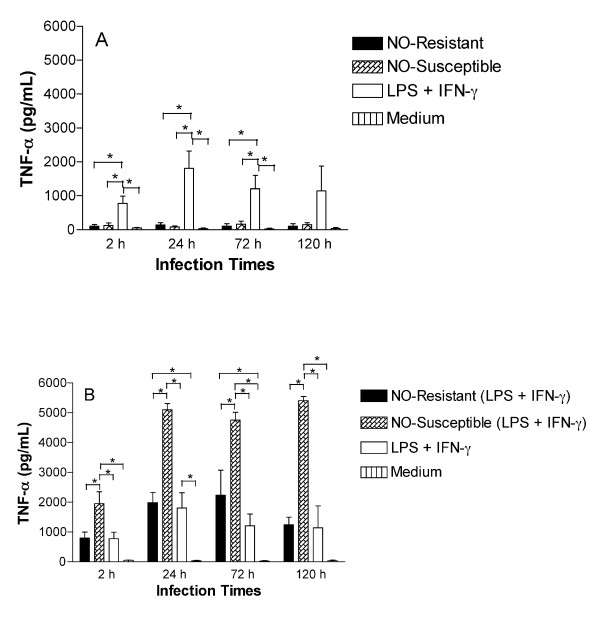
**TNF-α production is impaired in macrophages infected with NO-resistant *L. (V.) braziliensis***. Monocyte-derived macrophages were infected with NO-susceptible or NO-resistant *L. (V.) braziliensis*. After 2, 24, 72 or 120 h of infection the culture supernatants were harvest from non-stimulated macrophages (A) or macrophages treated with LPS (100 ng/mL) plus IFN-γ (10 ng/mL) thirty minutes prior to the addition of parasites (B). TNF-α levels were determined by ELISA. Each bar represents the mean ± SEM of three experiment from three separate donors (ANOVA, Tukey's multiple comparison Test, * = *p *< 0.05).

Non-stimulated macrophages did not produce TNF-α at a level that differed significantly from medium when they were infected with NO-susceptible and NO-resistant *L. (V.) braziliensis*. The positive control stimulation, LPS + IFN-γ, led to a significant increase of TNF-α production at all time points (Figure 4A). However, macrophages that were stimulated with LPS and IFN-γ 30 min prior to infection with NO-susceptible *L. (V.) braziliensis *produced significantly more TNF-α at all time points tested (2 h: 1954 ± 397; 24 h: 5101 ± 424; 72 h: 4766 ± 260; 120 h: 5407 ± 141), when compared to macrophages infected with NO-resistant isolates (2 h: 804 ± 193; 24 h: 1986 ± 347; 72 h: 2233 ± 842; 120 h: 1244 ± 252) or uninfected control (Figure 4B). The results are expressed as mean ± standard error of mean in pg/mL. These data suggest that NO-resistant *L. (V.) braziliensis *do not induce TNF-α production by macrophages whereas NO-susceptible isolates induces significant TNF-α production.

### IL-10 production is not altered in macrophages infected with *L (V) braziliensis* isolated from refractory patients

**Figure 5:**
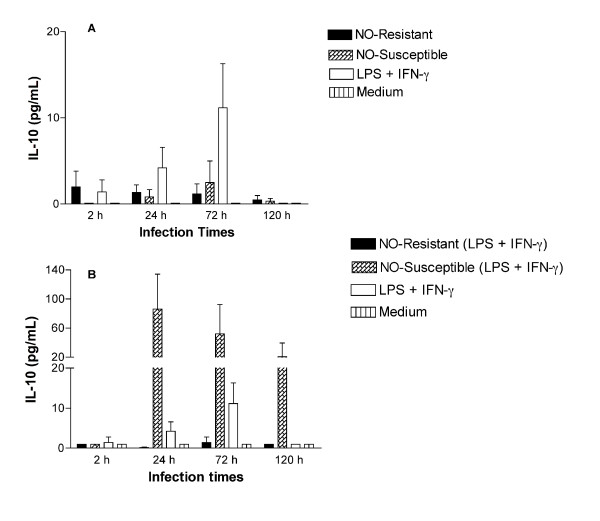
**IL-10 production not is altered in macrophages infected with isolated from refractory patients**. Monocyte-derived macrophages were infected with NO-susceptible and NO-resistant *L. (V.) braziliensis*. After 2, 24, 72 or 120 h of infection the culture supernatants of unstimulated macrophages (A) or from macrophages treated with LPS (100 ng/mL) plus IFN-γ (10 ng/mL) for thirty minutes before infection (B), were assessed for IL-10 levels by ELISA. Each bar represents the mean ± SEM infection levels in MDMs from three separate donors.

IL-10 is a cytokine that suppresses NO-production in macrophages. We therefore inquired whether the NO-resistant isolates suppressed TNF-α through enhanced IL-10 production. There were no statistically significant differences in IL-10 production in non-stimulated macrophages (Figure 5A). There was a trend toward increased IL-10 in the presence of LPS + IFN-γ 72 hrs after stimulation (11 ± 5 pg/mL), suggesting a secondary compensatory increase as has been observed in prior reports [25]. Paradoxically, in the constant presence of LPS + IFN-γ, macrophages infected with NO-susceptible *L. (V.) braziliensis *produced higher levels of IL-10 (24 h: 86 ± 48; 72 h: 52 ± 40; 120 h: 21 ± 19) than non-infected macrophages (1 pg/mL in all time points) or macrophages infected with NO-resistant *L. (V.) braziliensis *(approximately 1 pg/mL in all time points). However, statistically significant differences were not found (Figure 5B). These data suggest it is likely that another suppressor mechanism is responsible for the enhanced growth of NO-resistant parasites isolated from antimony refractory patients. It seems that these parasites do not activate macrophage to produce either TNF-α or IL-10, while NO-susceptible induce both cytokines.

## Discussion

Nitric oxide (NO) generated through the activity of the inducible nitric oxide synthase (iNOS) is a critical component of intracellular killing of *Leishmania *in murine macrophages and clearance of disease in mice [26,27]. More recently, the importance of NO in the microbial defenses of human macrophages against both *Leishmania *spp. and *Mycobacterium tuberculosis *has been suggested [28,29]. Recent data from our group demonstrated that NO resistance of human *L. (V.) braziliensis *isolates correlates directly with the initial size of the cutaneous lesion. Thus, patients infected with NO-resistant *Leishmania *present with a larger initial lesion size than patients infected with NO-resistant *Leishmania *[13]. *L. (V.) braziliensis *is a cause of cutaneous, mucosal and disseminated cutaneous leishmaniasis in endemic regions. Although some of these cutaneous lesions respond immediately to antimony treatment, other infections can be difficult to cure and require multiple courses of therapy [30,31].

We hypothesized that there may be inherent biological differences between the parasites isolates causing antimony-responsive versus antimony-refractory cutaneous lesions. The purpose of the current study was to evaluate phenotypic differences between *L. (V.) braziliensis *isolates from patients with localized cutaneous leishmaniasis who did or did not respond to antimony therapy.

Sensitivity of *L. (V.) braziliensis *parasite isolates to nitric oxide was measured using the MTT assay as a measure of viability after exposure to acidified NaNO_2_, a compound that releases NO and induces leishmanicidal activity [32]. We observed that more of the *L. (V.) braziliensis *isolates from antimony-refractory patients were NO resistant than *L. (V.) braziliensis *isolates derived from antimony-responsive patients. Furthermore, all the highly NO-resistant isolates were obtained from antimony-refractory patients.

Evaluation of human macrophage infections with NO-susceptible or NO-resistant isolates from either antimony-responsive or antimony-refractory patients also demonstrated that there are biological differences between the isolates, which correlate with NO susceptibility. NO-susceptible parasites exhibited transient initial growth in macrophages followed by intracellular killing, but only with a significant difference in NO-sensitive isolate from an antimony-responsive patient. The NO-resistant *L. (V.) braziliensis *isolates tested herein did not exhibit the initial growth phase or intracellular killing. Indeed, the highly NO-resistant isolate from an antimony-refractory patient displayed significant intracellular growth by 120 hrs of infection, even in the presence of LPS and IFN-γ, compounds that usually activate macrophages to become leishmanicidal [26,33]. The fact that both NO-susceptible and NO-resistant *L. (V.) braziliensis *isolates were maintained out to 120 hours of macrophage infection, even in the presence of LPS and IFN-γ (Figure 2), suggests that isolates can be resistant to other leishmanicidal mechanisms, including products of the NADPH oxidase (hydrogen peroxide, superoxide) [34-36].

It was previously reported that an isolate of *L. infantum *resistant to trivalent antimony (Sb^III^) *in vitro *was also resistant to an exogenous NO donor and to intracellular killing in activated macrophages [18]. Pentavalent antimony (Sb^V^) must be reduced to Sb^III ^to exhibit its leishmanicidal activity [18,19]. Sb^III ^in turn induces the production of reactive oxygen and nitrogen radicals in macrophages infected with *L. donovani *[20]. It follows logically that there may be a direct association between non-responsiveness to antimony therapy and NO resistance. Whether these isolates are also resistant to hydrogen peroxide and superoxide must still be tested. It has been demonstrated in *Leishmania donovani *that the parasite uses a cascade of enzymes that include cytosolic tryparedoxin peroxidase (cTXNPx) for the detoxification of peroxides, a mechanism necessary for the survival of digenetic parasites living in two disparate biological environments. The exposure of the parasite to a combination of H_2_O_2 _and nitric oxide resulted in a significant reduction of cTXNPx levels accompanied by high cell death. However, overexpression of cTXNPx by transfection increased the virulence, parasite burden in macrophages and resistance to clearance by antimony therapy, suggesting that differential expression of cTXNPx is linked to parasite resistance and virulence. [37].

The ability of *L. (V.) braziliensis *isolates to sustain infection may be related to their ability to resist NO toxicity [38]. Since arginine is a substrate of both iNOS and arginase, and the amount of arginine substrate is rate-limiting for both enzymes, consumption of arginine by the parasite can alter the activity of either enzyme in the intracellular environment. Arginase-mediated L-arginine hydrolysis produces polyamines which promote the growth of *Leishmania *[39,40], whereas arginine metabolism through NOS leads to toxic NO production. *L. amazonensis *produce nitric oxide synthase (NOS), at especially high levels in the axenic amastigote stage [41], and all *Leishmania *species studied make arginase [40]. The presence of either enzyme could deplete environmental arginine, generating either compartmentalized NO not available to kill *Leishmania *or generating polyamines that stimulate parasite growth [42]. Overactivity of either parasite enzyme would sequester arginine away from host. In addition to suppressing its synthesis through scavenging substrate, *Leishmania *may inhibit NO generation indirectly by inhibiting the macrophage synthesis of IL-12, decreasing the amount of IFN-γ produced by T and other cells, and in turn down-regulating the amount of NO produced through classical macrophage activation [43].

Using supernatants from culture of macrophages infected with NO-susceptible and NO-resistant *L. (V.) braziliensis*, we determined that macrophages stimulated with LPS and IFN-γ produced significantly lower amounts of TNF-α when they were infected with NO-resistant *L. (V.) braziliensis *than when they were infected with NO-susceptible *L. (V.) braziliensis *at all time points measured. This result suggests that NO-resistant *L. (V.) braziliensis *do not induce TNF-α production by macrophages.

TNF-α is a proinflammatory cytokine produced by macrophages and lymphocytes and it is important in the control of *Leishmania *spp. infections [11]. The impairment of TNF-α production by NO-resistant *L. (V.) braziliensis *is consistent with the finding that these isolates are present in patients with a severe form of tegumentary leishmaniasis [44].

*Leishmania *parasites can induce the production and/or secretion of various immunosuppressive signaling molecules including arachidonic acid metabolites and the cytokines TGF-β and IL-10 [45]. IL-10 in turn suppresses IFN-γ and TNF-α, and consequently inhibits NO-production by macrophages [46]. As a first step in examining mechanisms of TNF-α suppression, we examined whether IL-10 was induced by NO-resistant *L. (V.) braziliensis. *Disproving our hypothesis, we observed minimal IL-10 production by non-stimulated macrophages infected with NO-susceptible and NO-resistant *L. (V.) braziliensis*. Paradoxically, when macrophages were stimulated with LPS and IFN-γ and infected with NO-susceptible *L. (V.) braziliensis *they produced high levels of IL-10, whereas macrophages infected with NO-resistant *L. (V.) braziliensis *produced minimal. We interpret this as a compensatory increase in IL-10 in response to the increased type 1 cytokines induced, illustrated by the TNF-α increase observed in Figure 4. These results show that enhanced IL-10 is not the mechanism leading to absence of TNF-α production in macrophages infected with NO-resistant *L. (V.) braziliensis*. It is likely that another mechanism generates the immunosuppressive signal delivered by the resistant parasites. Candidates would include PGE2, which favors parasite intracellular survival by suppressing TNF-α, IL-1 and reactive oxygen intermediates [47], and the cytokine TGF-β whose production correlates with diminished iNOS expression [48-52].

## Conclusion

The data presented in this report suggest that resistance of human *L. (V.) braziliensis *isolates to NO is associated with nonresponsiveness to antimony therapy and an impaired macrophage activation to produce TNF-α. Together with our previously published data indicating that NO resistance is associated with a worsened clinical presentation of American Tegumentary Leishmaniasis, these data suggest there may be consequences of NO resistance relevant to human disease. These observations may serve as a basis to test clinical isolates for resistance to toxic oxidants as a predictor of the course and drug-responsiveness of disease.

## Competing interests

The authors declare that they have no competing interests.

## Authors' contributions

AS, AG, JMBP, EMC and RPA participated equally in the study design. AS, AG, JMBP and RPA performed all the parasites experiments. AS, AG, ARJ, TRM, MEW and RPA drafted manuscript. AS, AG and JMBP participated in the experiments of human macrophages infection and cytokine detection. LHG, ARJ, RPA and EMC participated of the procedure of parasite achievement and clinical exams at Corte de Pedra, Bahia, Brazil. All authors read and approved the final manuscript.

## Pre-publication history

The pre-publication history for this paper can be accessed here:

http://www.biomedcentral.com/1471-2334/10/209/prepub
